# Structural Differences between the Avian and Human H7N9 Hemagglutinin Proteins Are Attributable to Modifications in Salt Bridge Formation: A Computational Study with Implications in Viral Evolution

**DOI:** 10.1371/journal.pone.0076764

**Published:** 2013-10-07

**Authors:** Marni E. Cueno, Kenichi Imai, Muneaki Tamura, Kuniyasu Ochiai

**Affiliations:** Department of Microbiology, Nihon University School of Dentistry, Tokyo, Japan; Boston University School of Medicine, United States of America

## Abstract

Influenza A hemagglutinin (HA) is a homotrimeric glycoprotein composed of a fibrous globular stem supporting a globular head containing three sialic acid binding sites responsible for infection. The H7N9 strain has consistently infected an avian host, however, the novel 2013 strain is now capable of infecting a human host which would imply that the HA in both strains structurally differ. A better understanding of the structural differences between the avian and human H7N9 strains may shed light into viral evolution and transmissibility. In this study, we elucidated the structural differences between the avian and human H7N9 strains. Throughout the study, we generated HA homology models, verified the quality of each model, superimposed HA homology models to determine structural differences, and, likewise, elucidated the probable cause for these structural differences. We detected two different types of structural differences between the novel H7N9 human and representative avian strains, wherein, one type (Pattern-1) showed three non-overlapping regions while the other type (Pattern-2) showed only one non-overlapping region. In addition, we found that superimposed HA homology models exhibiting Pattern-1 contain three non-overlapping regions designated as: Region-1 (S157_1_-A160_1_); Region-3 (R262_1_-S265_1_); and Region-4 (S270_1_-D281_1_), whereas, superimposed HA homology models showing Pattern-2 only contain one non-overlapping region designated as Region-2 (S137_1_-S145_1_). We attributed the two patterns we observed to either the presence of salt bridges involving the E114_1_ residue or absence of the R141_1_:D77_1_ salt bridge. Interestingly, comparison between the human H7N7 and H7N9 HA homology models showed high structural similarity. We propose that the putative absence of the R141_1_:D77_1_ salt bridge coupled with the putative presence of the E114_1_:R262_1_ and E114_1_:K264_1_ salt bridges found in the 2013 H7N9 HA homology model is associated to human-type receptor binding. This highlights the possible significance of HA salt bridge formation modifications in viral infectivity, immune escape, transmissibility and evolution.

## Introduction

Influenza A virus is an RNA virus that initiates infection primarily attributed to the hemagglutinin (HA) glycoprotein. HA is a homotrimeric glycoprotein comprising of a fibrous globular stem inserted into the viral membrane supporting a globular head containing three sialic acid binding sites [1]–[3]. Each HA protomer can be divided into two polypeptides, HA1 and HA2, generated from a single nascent peptide chain through protease cleavage [1], [4]. The membrane-distal domain (HA1) can be further divided into the receptor-binding and vestigial esterase subdomains while the stem region is composed of an F fusion subdomain (HA2) and both the N- and C-terminal segments of an F’ fusion subdomain (HA1) [5].

Influenza infection and transmission involves HA binding to sialic acids found in the cell surface [6], [7] and, in turn, can determine what host can be infected [8]. HA receptor-binding is influenced by several factors such as glycosylation [1], neutralizing antibodies [1], and inter-residue atomic interactions [9]. Moreover, HA receptor-binding avidity has been correlated to HA antigenic drift [10] which is hypothesized to, subsequently, influence neuraminidase antigenic drift [11]. This highlights the importance of the HA receptor-binding in viral infectivity, immune escape, transmissibility and evolution [6], [8], [10], [12].

Human infections attributable to an avian influenza virus have thus far been limited to the H5, H7, and H9 subtypes [13]–[18]. Among the H7 subtypes, previously recorded avian-to-human transmissions were observed in the Netherlands (2003 H7N7 strain) [15] and Canada (2004 H7N3 strain) [19]. The novel 2013 H7N9 strain is the first H7 subtype recorded in Asia that was transmitted to humans [20] and its protein structure compared to previous H7N9 avian strains has not been elucidated. A better understanding of the structural changes that occurred in the 2013 H7N9 strain as compared to previous strains may shed light into viral evolution and transmissibility. In addition, this may assist in designing vaccines and other antiviral therapies.

Here, we superimposed the 2013 H7N9 HA homology generated from a human strain with those from previous avian strains. We detected two different types of structural differences, wherein, one type showed three non-overlapping regions while the other type showed only one non-overlapping region. Moreover, we proposed that these structural differences are associated with the presence and absence of salt bridge formation, respectively.

## Methods

### Data mining

H7N9 HA amino acid sequences were collected from the National Center for Biological Information website. HA amino acid sequences obtained and analyzed were from avian-infecting strains (1988, 1995, 2000, 2006, 2008a, 2008b, 2009, 2011) and a novel human-infecting strain (2013). Note that there were two different 2008 strains. In this study, we used the following representative amino acid sequences: novel 2013 human strain (Genebank accession: AGI60301), representative 2011 (Genebank accession: AFV33947) and 2008b (Genebank accession: BAH22785) avian strains. Similarly, we compared the HA homology models of the 2003 H7N7 human strain (Genebank accession: AAR02640) with those from the representative 2013 H7N9 human and 2011 H7N9 avian strains to distinguish any structural differences between the two H7 subtypes. We followed the numbering scheme as previously published [21] and we designated subscript 1 and 2 to refer to HA_1_ and HA_2_, respectively.

### Homology modeling and model quality estimation

We predicted the protein structure using the Phyre server [22]. The most recent version (Phyre2) was utilized in this study. Briefly, the Phyre server uses a library of known protein structures taken from the SCOP database and augmented with newer depositions in the PDB database. The sequence of each predicted 3D structure is scanned against a non-redundant sequence database and the top ten highest scoring alignments are then used to construct full 3D models.

Qualitative Model Energy Analyses (QMEAN) scores were determined to estimate the absolute quality of each homology model generated [23], [24]. QMEAN scoring function is based on the linear combination of six structural descriptors and reflects the predicted global model reliability ranging from 0 to 1 where values close to 1 are considered reliable whereas values close to 0 are considered unreliable.

### Model superimposition

SuperPose [25] was used to superimpose all H7N9 avian strains for comparison and, similarly, to determine any differences between the novel 2013 and representative 2011 HA homology models. Briefly, SuperPose is composed of two parts: (1) a front-end web interface which is written in Perl and HTML; and (2) a back-end for alignment, superposition, Root Mean Square Deviation (RMSD) calculation and rendering which is written in Perl and C. We superimposed the all avian HA homology models with the 2013 HA homology model. Representative superimpositions using the 2008b and 2011, 2011 and 2013, and 2008 and 2013 H7N9 HA homology models were used to determine the structural differences between HA homology models and, likewise, RMSD scores of the superimposed Cα backbone. RMSD scores close to 0 would insinuate low structural difference between the homology models.

### Structural analyses

We analyzed the predicted protein models using the Jmol software [26]. Briefly, Jmol is a free open source applet developed for the interactive display of three-dimensional chemical structures which also have features for biomolecules. In addition to reading molecular models, Jmol can also read script files and allow instructions or commands to be applied to the predicted model. The ribbon structure of the HA homology models were used for structural analyses and amino acid residues of interest were changed into wireframe conformations. The van der Waals interactions of specific amino acid residues were also considered when appropriate. In addition, Jmol was also used to determine all distance measurements made between amino acid residues of interest. All distance measurements made are in Å.

## Results

### H7N9 HA homology models generated are reliable

**Figure 1:**
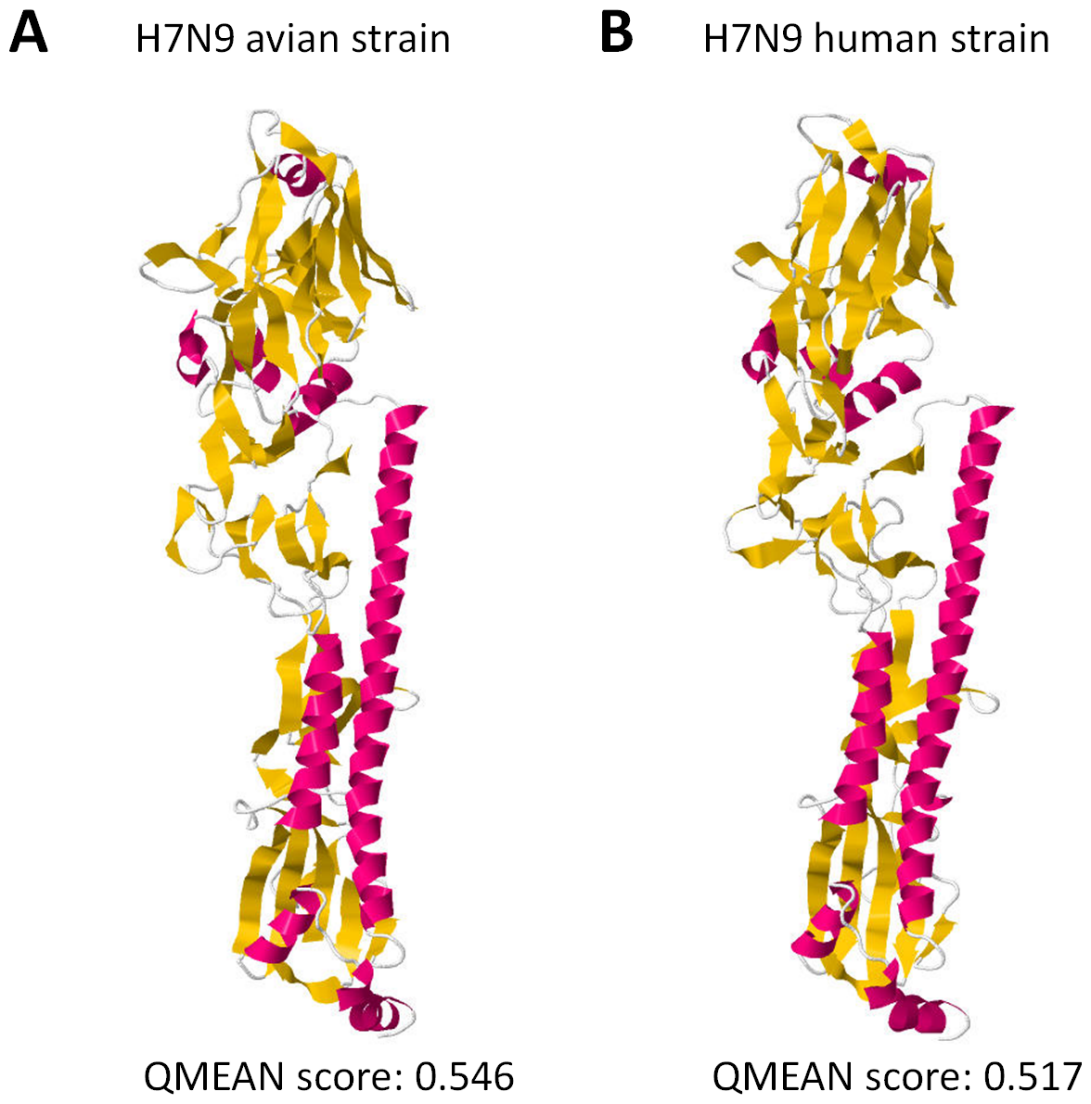
Quality estimation of influenza A H7N9 hemagglutinin homology models generated. Ribbon structure and model quality estimation of a (A) representative avian and (B) novel human H7N9 HA homology models. QMEAN score is indicated below. QMEAN scores > 0.5 are considered reliable. α-helix (red), ß-sheet (yellow), and structural loops (white) are indicated.

In producing experimental or theoretical models of protein structures, it is always important to assess model accuracy and reliability before proceeding to further analyses [27]. To confirm the quality of each HA homology model generated and establish structural differences, we determined QMEAN scores of all HA homology models analyzed. As seen in Figures 1A and 1B, both HA homology models of the avian and human H7N9 strains have QMEAN scores >0.5 implying that the homology models are reliable for further analyses.

### 2013 HA homology model has structural differences with avian HA homology models

**Figure 2:**
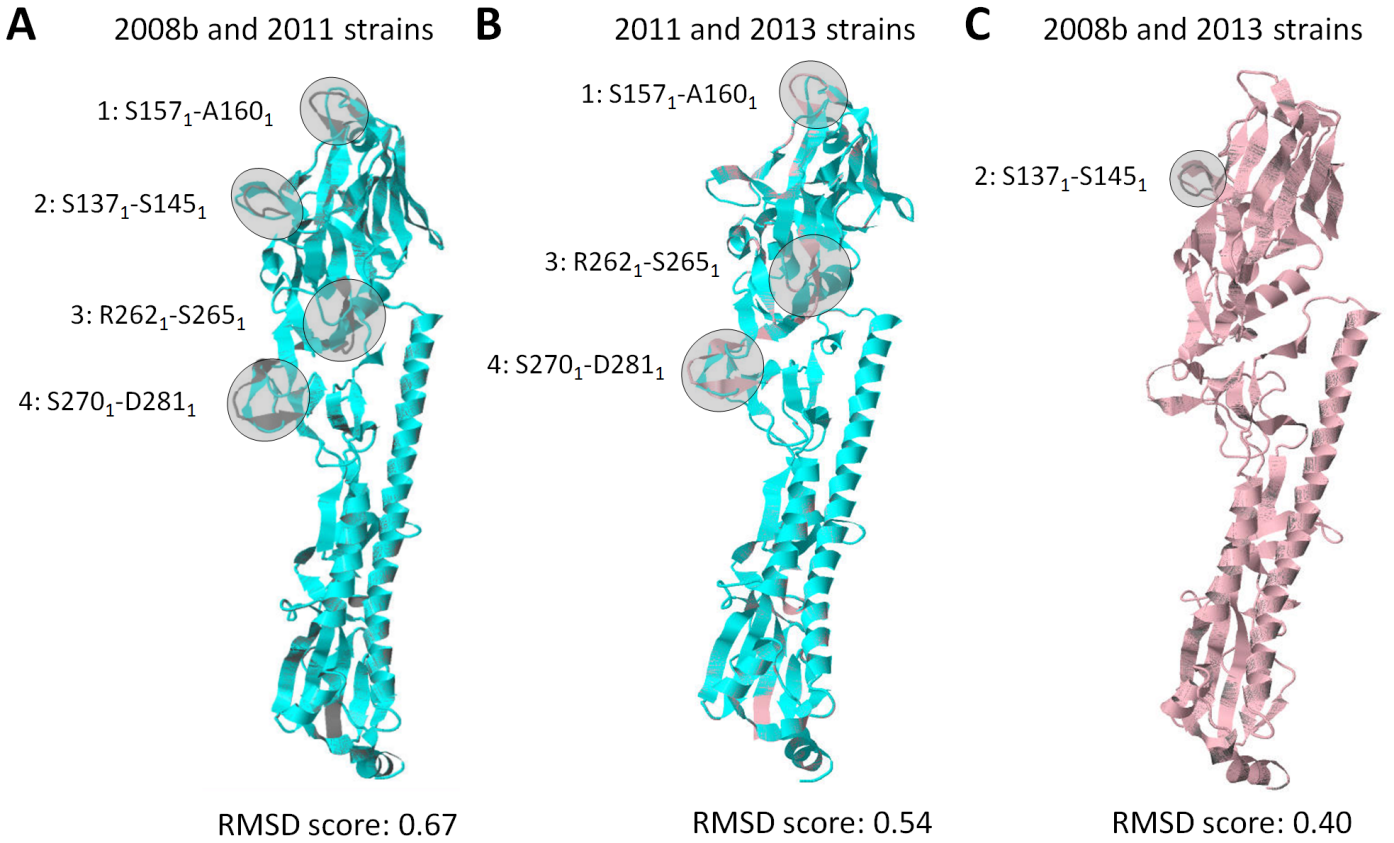
H7N9 human HA homology model has two patterns of structural differences with avian HA homology models. H7N9 HA homology model superimposition of the (A) 2008b and 2011 strains, (B) 2011 and 2013 strains, and (C) 2008b and 2013 strains. HA homology models of the 2008b (gray), 2011 (blue), and 2013 (pink) strains are shown. Non-overlapping regions representing structural differences (shaded in gray) are indicated. RMSD scores of the superimposed Cα backbone are indicated below. RMSD scores close to 0 would insinuate low structural difference between the homology models.

To establish the structural differences between the HA of the human and avian strains, HA homology model superimposition was performed and representative superimpositions using the 2008b and 2011, 2011 and 2013, and 2008b and 2013 HA homology models were shown. In all superimpositions made, we found that superimposed models with more structural differences have higher RMSD scores. Moreover, superimposition of the 2008b and 2011 HA homology models showed four non-overlapping regions designated as: Region-1 (S157_1_-A160_1_); Region-2 (S137_1_-S145_1_); Region-3 (R262_1_-S265_1_); and Region-4 (S270_1_-D281_1_) (Fig. 2A) suggesting that among avian strains, there are structural differences in the HA homology models. Superimposition of the 2013 HA homology model showed two distinct patterns of HA structural differences, whereby, Pattern-1 only has three non-overlapping regions previously designated as Region-1, Region-3, and Region-4 (Fig. 2B), whereas, Pattern-2 only has one non-overlapping region previously designated as Region-2 (Fig. 2C). In addition, we detected Pattern-1 when the 2013 HA homology model was superimposed with the 1988, 1995, 2000, 2006, 2008a, or 2011 HA homology models studied, whereas, Pattern-2 was detected when the 2013 HA homology model was superimposed with the 2002, 2008b, or 2009 HA homology models (data shown using representative strains) insinuating that the observed patterns of structural differences are consistent. Moreover, this would suggest the 2013 HA homology model is structurally more related to the 2002, 2008b, and 2009 models.

### Pattern-1 is ascribable to salt bridge formation involving the E114_1_ residue

**Figure 3:**
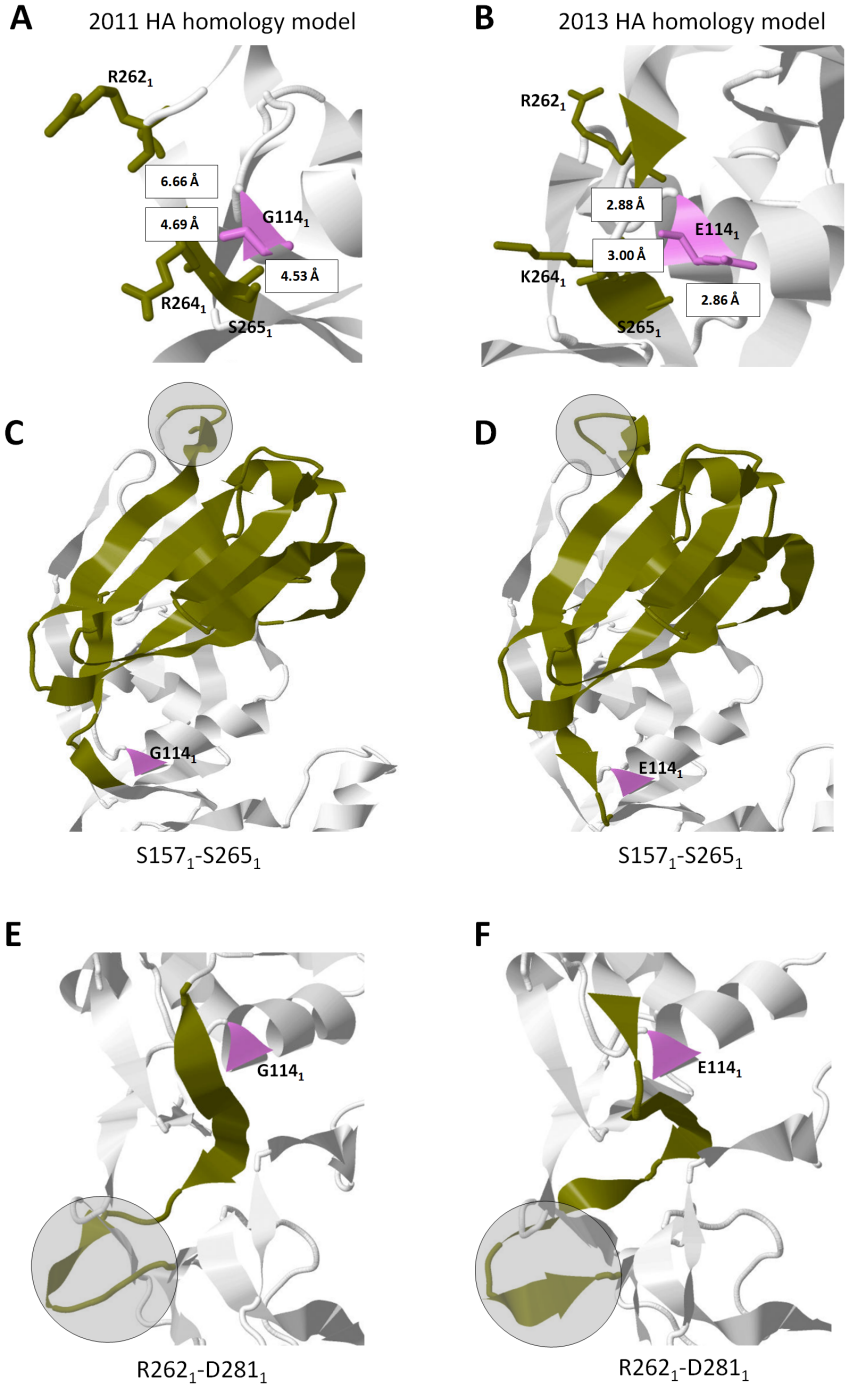
Pattern-1 structural differences are ascribable to salt bridge formation involving amino acid residue 114_1_. Distance measurements of amino acid residues 262_1_, 264_1_, and 265_1_ (green) relative to residue 114_1_ (violet) found in the (A) 2011 and (B) 2013 HA homology models. All amino acid residues indicated are in a wireframe structure. All measurements are indicated in Å. Interrelationship of HA structural differences (shaded in gray) observed in the (C,E) 2011 and (D,F) 2013 HA homology models are highlighted in green. Amino acid residue 114_1_ is indicated in violet.

To elucidate the structural differences observed in Pattern-1, we looked into the neighboring strands of each non-overlapping region (Region-1, -2, and -3), identified amino acid residue/s and its structural properties that vary between the superimposed HA homology models, and, subsequently, determine its putative effects on the non-overlapping region/s. As shown in Figure 3A and 3B, we found that in Region-3, the neighboring amino acid residue 114_1_ differs between the 2011 (G114_1_) and 2013 (E114_1_) HA homology models. In addition, using three amino acid residues (253_1_, 255_1_, 256_1_) as a reference point, we found that the neighboring R262_1_-S265_1_ strand is farther in the 2011 HA homology model (> 4.0 Å) as compared to the 2013 model (< 4.0 Å). All three structural differences (in the form of non-overlapping regions) are located within HA1 and, in particular, Region-3 is located between Region-1 and -4. This would imply that Region-3 is structurally interrelated to both Region-1 and -4.

To establish the possible structural link between the three non-overlapping regions, we retraced the strands in-between each region and found that Region-3 can be structurally linked to both Region-1 (Figs. 3C and 3D) and Region-4 (Figs. 3E and 3F). It is worth mentioning that the G114_1_ residue was found in the 1988, 1995, 2000, 2006, 2008a, and 2011 HA homology models, whereas, the E114_1_ residue was found in the 2002, 2008b, and 2009 HA homology models (data not shown).

### Pattern-2 is influenced by the absence of the R141_1_:D77_1_ salt bridge

**Figure 4:**
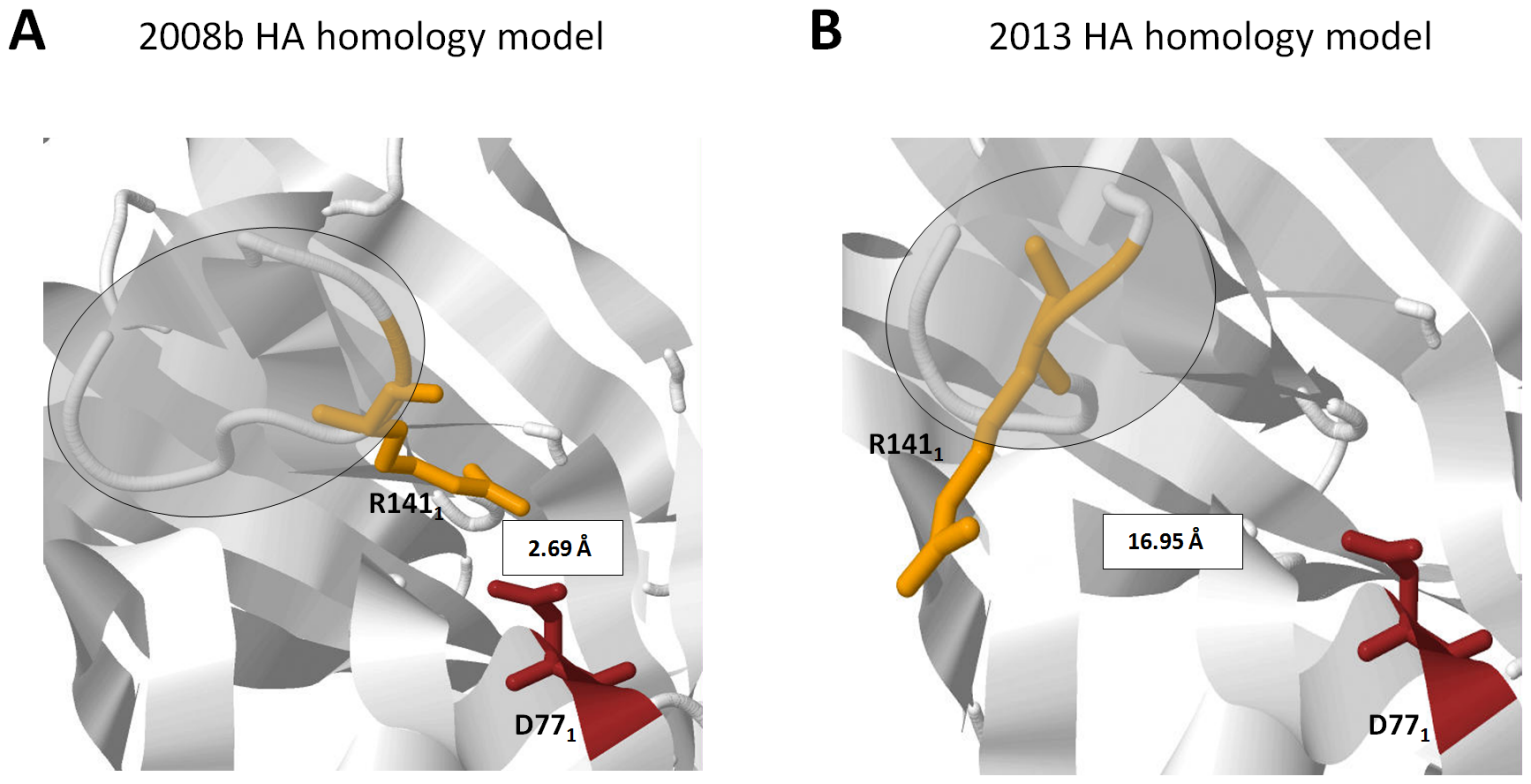
Pattern-2 structural difference is associated with the absence of the R141_1_:D77_1_ salt bridge. Distance measurement between R141_1_ (orange) and D77_1_ (maroon) found in the (A) 2008b and (B) 2013 HA homology models. All amino acid residues indicated are in a wireframe structure. All measurements are indicated in Å. HA structural difference is shaded in gray.

To expound on the structural difference observed in Pattern-2, we looked into the neighboring strands of Region-2, identified amino acid residue/s and its structural properties that vary between the superimposed HA homology models, and, subsequently, determine its putative effects on Region-2. We identified no amino acid changes within neighboring strands, however, we found a difference in the distance measurements between the S137_1_-S145_1_ strand (using R141_1_ as a reference point) and its neighboring strand containing the D77_1_ residue, wherein, the 2008 HA homology model is closer (< 4.0 Å) as compared to the 2013 strain (> 4.0 Å) as seen in Figures 4A and 4B, respectively.

### 2003 H7N7 and 2013 H7N9 HA homology models have high structural similarities

**Figure 5:**
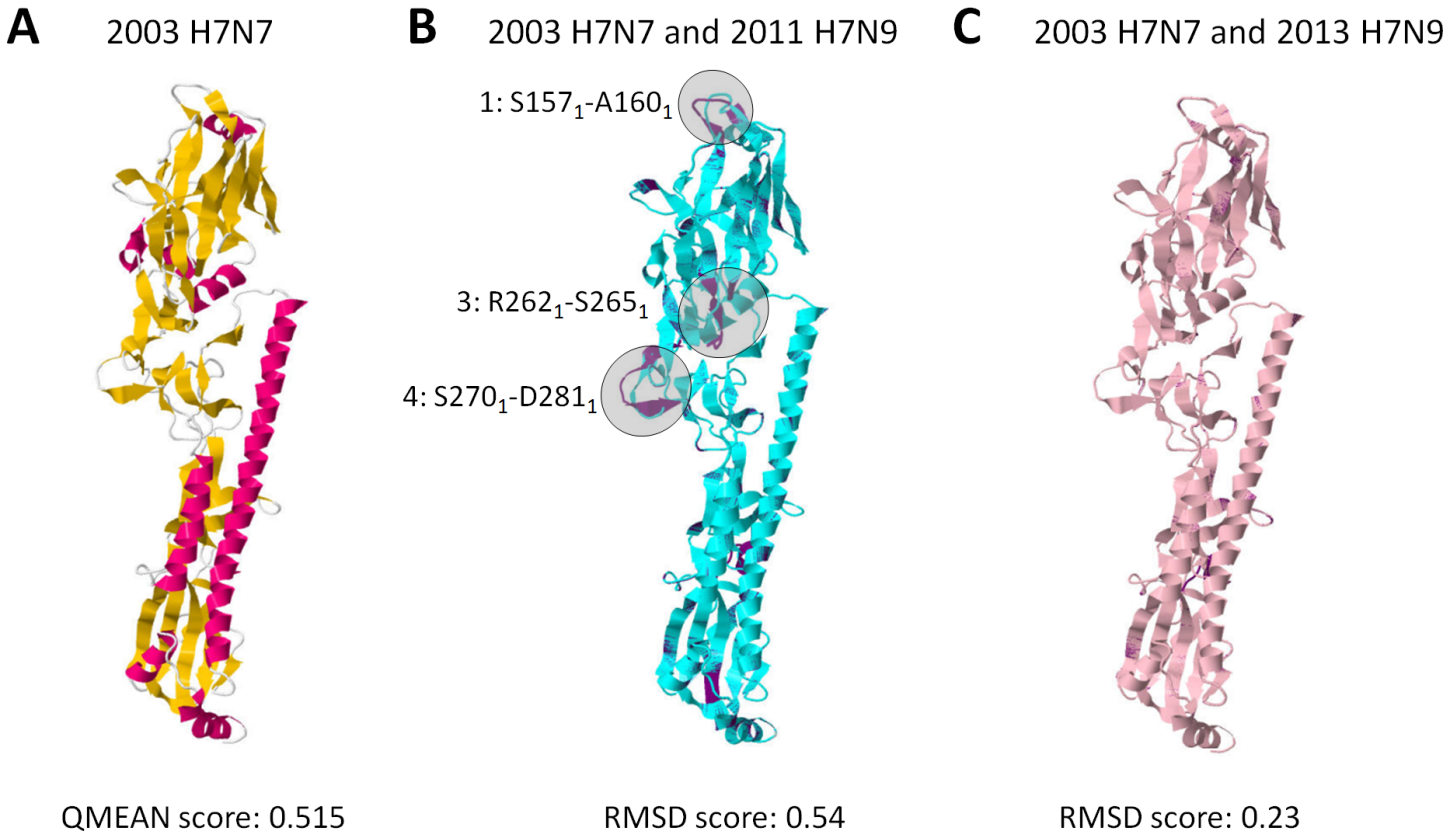
HA homology models of the 2003 H7N7 and 2013 H7N9 human strains are structurally similar. (A) Ribbon structure and model quality estimation of a representative 2003 H7N7 human HA homology model. QMEAN score is indicated below. QMEAN scores > 0.5 are considered reliable. α-helix (red), ß-sheet (yellow), and structural loops (white) are indicated. HA homology model superimposition of the representative (B) 2003 H7N7 human and 2011 H7N9 avian strains, and (C) 2003 H7N7 and 2013 H7N9 human strains. HA homology models of the 2003 H7N7 (violet), 2011 H7N9 (blue), and 2013 (pink) strains are shown. Non-overlapping regions representing structural differences (shaded in gray) are indicated. RMSD scores of the superimposed Cα backbone are indicated below. RMSD scores close to 0 would insinuate low structural difference between the homology models.

To distinguish any structural differences between the 2003 H7N7 and 2013 H7N9 HA homology models, we generated the 2003 H7N7 HA homology model, verified its quality, and, subsequently, compared it to HA homology models of representative 2011 avian and 2013 human strains through superimposition. As seen in Figure 5A, HA homology model of the 2003 human H7N7 strain has a QMEAN score >0.5 implying that the homology models are reliable for further analyses. Interestingly, HA homology model superimposition of the 2003 H7N7 human and 2013 H7N9 avian strains showed a Pattern-1 structural difference (Fig. 5B) while superimposition with the 2013 H7N9 human strain showed very high structural similarities.

## Discussion

Throughout this study we identified two patterns of structural differences observed in the superimposed HA homology models of the novel H7N9 human and representative avian strains. We attributed these two patterns to either the presence or absence of salt bridges. A salt bridge is a combination of two non-covalent interactions (hydrogen bonding and electrostatic interactions) and, in the influenza HA, salt bridges are commonly affected by pH [28]–[31]. The most common salt bridges occur from the anionic carboxylate of either aspartic acid or glutamic acid and the cationic ammonium from lysine or the guanidinium of arginine [29].

In the superimposed HA homology model exhibiting Pattern-1, we hypothesize that the non-overlapping regions (Region-1, -3, and -4) found in the superimposed HA homology model is associated to the amino acid difference at residue 114_1_, wherein, a G114_1_ residue does not favor salt bridge formation with a neighboring strand (in this case the R262_1_-S265_1_ strand) while a E114_1_ residue favors salt bridge formation. Glutamic acid (or Glutamate) is a negatively charged polar amino acid that prefers to be exposed to the aqueous environment and when found buried within a protein can form salt bridges if the distance requirement (< 4.0 Å) is satisfied [29]. We propose that both the structural differences observed in Pattern-1 and the probable interrelationships within the three non-overlapping regions are attributable to the putative E114_1_:R262_1_ and E114_1_:K264_1_ salt bridges found in Region-3. In the superimposed HA homology model showing Pattern-2, we found that the difference in distance between residues R141_1_ and D77_1_ affected salt bridge formation. This would indicate that the 2008b HA homology model has an R141_1_:D77_1_ salt bridge, whereas, that of the 2013 model has no R141_1_:D77_1_ salt bridge.

Presence or absence of salt bridge formation can structurally influence the protein backbone and, subsequently, affect overall protein function [29], [32], [33]. In the past, H7N9 strains only infected avian hosts, however, with the novel 2013 strain, human infection is now made possible [20] insinuating that changes occurred in the HA1 receptor-binding domain (RBD). The HA1 RBD play a significant role in several viral activities and changes in the RBD structural property would have a crucial impact on viral HA function [1], [10], [12], [28], [34]–[37]. In Pattern-1, the 2013 HA homology model is structurally different in Regions-1, -3, and -4 while being structurally similar in Region-2, whereas, in Pattern-2, the 2013 HA homology model is structurally different in Region-2 while being structurally similar in Regions-1, -3, and -4.

Interestingly, residues that were previously published to improve viral binding to human-type receptors [38], [39] are found within the structural differences we observed. We suspect that these structural differences contributed to the 2013 H7N9 host shift. Moreover, a high structural similarity between the HA homology models of the 2003 H7N7 and 2013 H7N9 human strains would further insinuate that the structural conformation adapted by the novel 2013 H7N9 strain, ascribable to our observed difference in salt bridge formation, favor the human-type receptor binding. Thus, we hypothesize that the putative absence of the R141_1_:D77_1_ salt bridge coupled with the putative presence of the E114_1_:R262_1_ and E114_1_:K264_1_ salt bridges found in the 2013 HA homology model contributed to the host shift which allowed for the viral binding to human-type receptors. Admittedly, additional experimentation is required to further prove these points.

In conclusion, we identified two patterns of structural differences in the superimposed HA homology models between the novel H7N9 human and representative avian strains. In addition, we found that superimposed HA homology models exhibiting Pattern-1 contain three non-overlapping regions (Region-1, -3, and -4), whereas, superimposed HA homology models showing Pattern-2 only contain one non-overlapping region (Region-2). We attributed the two patterns we observed to either the presence or absence of salt bridges. Moreover, we propose that the absence of the R141_1_:D77_1_ salt bridge coupled with the presence of the E114_1_:R262_1_ and E114_1_:K264_1_ salt bridges found in the 2013 HA homology model lead to a high structural similarity found between the HA homology models of the 2003 H7N7 and 2013 H7N9 human strains which, subsequently, allowed for viral binding to human-type receptors. Furthermore, this would insinuate that modifications in HA salt bridge formation may consequentially influence viral infectivity, immune escape, transmissibility and evolution.
